# Virtual, Augmented, and Mixed Reality for Motor Neurorehabilitation: Scoping Review Focused on the Role of Body Representation

**DOI:** 10.2196/63487

**Published:** 2025-12-17

**Authors:** Massimo Magrini, Olivia Curzio, Cristina Dolciotti, Gabriele Donzelli, Maria Cristina Imiotti, Fabrizio Minichilli, Davide Moroni, Paolo Bongioanni

**Affiliations:** 1 Institute of Information Science and Technologies, Alessandro Faedo, National Research Council, Pisa, Italy; 2 Institute of Clinical Physiology, National Research Council, Via Moruzzi 1, Pisa, 56124, Italy, 39 050-3152105, 39 050-3152095; 3 Spinal Cord Injuries Unit, Neuroscience Department, Pisa University Hospital, Pisa, Italy

**Keywords:** body representation, embodiment cognition, virtual reality, augmented reality, neurorehabilitation

## Abstract

**Background:**

Extended reality (XR), encompassing virtual reality, augmented reality (AR), and mixed reality, is increasingly being used in neurorehabilitation to provide multisensory feedback and promote neural plasticity in sensorimotor networks.

**Objective:**

This scoping review aimed to (1) examine how XR technologies are applied in motor neurorehabilitation, (2) explore how body representation and somatic embodiment are addressed, and (3) analyze the methodological designs of XR-based interventions.

**Methods:**

This review was conducted in accordance with the PRISMA-ScR (Preferred Reporting Items for Systematic Reviews and Meta-Analyses extension for Scoping Reviews) guidelines, with a comprehensive search across PubMed, Embase, Scopus, and Web of Science from inception to December 2023. Eligible studies included original research involving XR-based interventions explicitly targeting neurorehabilitation. Studies related to somatic embodiment and reporting data on implementation and user outcomes were considered without date restriction. Three independent reviewers conducted screening in Covidence. The following variables were extracted: study design, participant characteristics, XR devices and software, experimentation details, treatment approaches, and evaluation methods. Methodological quality of the included studies was assessed using the Newcastle-Ottawa Scale and the Murad Scale. Findings have been presented in tabular and narrative formats.

**Results:**

Twenty-six studies met the inclusion criteria, and these were mainly clinical trials involving patients with neurological conditions, particularly poststroke status (n=6) and spinal cord injury (n=2). Several studies provided physiological data, including electroencephalography (n=12), electromyography (n=2), magnetic resonance imaging (n=1), galvanic skin response (n=1), electrodermal activity (n=1), and motor-evoked potential data (n=1). Two studies used noninvasive brain stimulation, and another two used eye tracking. Most studies (n=17) used built-in motion sensors; however, some (n=8) analyzed the data quantitatively. Unity 3D was the most frequently used development platform (n=8). First-person (n=20) and third-person (n=2) perspectives were used, and 4 studies combined both perspectives. Interventions mainly targeted sensorimotor deficits, with improvements in motor and cognitive performance. Sixteen studies addressed body perception, focusing on limb embodiment. Questionnaires were the most frequently used evaluation tools (n=18), and 3 studies used standardized tests. Some studies (n=7) investigated body ownership under visuomotor inconsistencies with or without visuotactile stimulation. XR was primarily applied to enhance sensorimotor recovery and assess device feasibility. Few studies directly measured embodiment (n=4), ownership (n=2), or self-location (n=2). The ability of XR platforms to deliver multisensory feedback appears to facilitate sensorimotor learning and support a more accurate body schema.

**Conclusions:**

Evidence from the studies supports the usefulness of XR in enhancing reinforcement learning and facilitating recovery in neurorehabilitation. Tailored XR approaches, which are grounded in embodiment principles and patient-specific needs, show promise for improving outcomes in neurological rehabilitation programs. The AR paradigm, which could offer several advantages, was not explored in depth, perhaps due to its difficult implementation during the period considered.

## Introduction

### Background

Motor neurorehabilitation plays an important role in restoring movement following neurological injuries, such as stroke, spinal cord injury (SCI), and traumatic brain injury. Extended reality (XR), including virtual reality (VR), augmented reality (AR), and mixed reality (MR), has emerged in recent years as a promising tool to enhance rehabilitation by providing immersive, interactive, and customizable therapeutic environments [1]. Regarding the integration of XR in motor neurorehabilitation, questions remain about its usability and implementation in clinical practice. These immersive technologies offer possibilities for engaging patients in enriched environments that can simulate real-world motor activities and provide useful real-time feedback [2]. XR applications can support sensorimotor learning by leveraging visual, proprioceptive, and tactile cues [3]. A growing body of research has begun to explore how XR technologies can influence body representation, a key neurocognitive construct underlying motor control and recovery. Concepts, such as body schema, body image, and embodiment, are recognized as relevant to rehabilitation outcomes, especially in interventions that aim to restore motor function by altering or reinforcing body-related perceptual and sensorimotor processes [4,5]. Despite promising developments, there is a lack of methodological clarity in how XR-based interventions for motor neurorehabilitation conceptualize and implement body representation.

### Contribution of the Sensorimotor System, Motor Imagery, and Cognitive Domains to Body Representation

In scientific terms, body representation comprises 2 primary modes of expression, both of which are internal to the subject: body schema and body image. Although both modes are internal to the subject, body schema can be defined as an objective representation because it is conditioned by multisensory perception, whereas body image is a subjective representation resulting from cognitive processing and emotional response [6].

While body schema has been extensively studied in relation to motor and postural outcomes or adaptations following neurological diseases, body image, referring to the subjective mental representation of one’s own body, has involved the study of complex neural pathways that create a synthesis between the objective and subjective representations of the body [7].

Neuroimaging studies have identified specialized cortical areas for processing body shapes and actions, highlighting the significance of interoception alongside exteroception and proprioception for body awareness. There is evidence that the insular cortex processes interoceptive signals, thus playing a key role in determining body consciousness [8].

Despite extensive research on the integrated representation of the body, precise brain structures remain elusive. However, the extra striate body area has emerged as a neural substrate for body shape and size perception [9]. In this regard, it is worth noting that many important seminal papers on brain imaging related to body schema and body image have been produced overall and specifically in the multisensory neurorehabilitation field [6,10-14].

Proprioception, a key sensorimotor system modality, enables individuals to perceive body position and movement independently of vision. Additionally, the discovery of sensory neurons in frontal motor circuits challenges the traditional view of motor control, suggesting sensorimotor system involvement in cognitive processes [15]. The visual perception of the body and its movement involves other considerations that refer to the so-called mirror neurons. Mirror neurons, which are a part of the sensorimotor system, activate during both action execution and observation, fostering a sense of embodiment [16]. Besides proprioception, the sensorimotor system integrates information from other senses like vision and touch, enhancing body representation accuracy [17-19]. Motor skill learning alters body perception and control, and it is influenced by top-down factors and task demands [20]. Experimental findings suggest that mediated sensory perception, like mirrored hand movements, can induce abnormal body representations [19]. When sensory or cognitive deficits limit body movement in space, the contribution of motor imagery becomes important. Motor imagery is vital for cognitive body representation and is used in contexts such as rehabilitation to enhance motor skills and awareness [21]. Proprioceptive input significantly influences mental rotation, highlighting its role in motor control and spatial awareness [22].

Cognitive processes, including memory and attention, shape body representation and awareness. Brain regions like the parietal cortex integrate sensory inputs to create coherent body representations [23]. Subcortical structures like the limbic system regulate emotional responses and body memory, while brain plasticity modifies body image in response to experiences [24]. The peripersonal space surrounding the body plays a crucial role in physical interactions and self-location. The peripersonal space adapts based on experiences, technology, and social interactions, supporting bodily self-consciousness and higher-level cognition [25].

Body image construction involves emotional regulation and cognitive processes. Stress can alter body perception, while self-esteem can enhance it [26], and body dissatisfaction is influenced by factors like visual memory and inhibition [27]. Changes in body schema and distortions in body image can result from various neurological and psychiatric conditions [28]. For instance, individuals with SCI or anesthesia may continue to experience sensations related to body size, shape, and posture despite the absence of immediate sensory signals, suggesting the role of cognitive and emotional processing in body image [29]. Moreover, studies exploring body image experiences in people with SCI have revealed categories such as physical appearance concerns, negative functional features, and body disconnection [30]. Similarly, in patients with multiple sclerosis, higher disability levels are associated with more negative body perceptions and lower self-esteem [31].

In conditions like stroke, damage to brain areas involved in body perception and sensory processing can lead to neglect syndrome, where individuals may ignore or be unaware of one side of their body [31,32]. Moreover, phenomena like phantom limb syndrome, which is observed in amputees, and alien limb syndrome, which is seen in conditions like corticobasal degeneration, illustrate how the brain’s representation of the body can persist despite physical changes or dysfunction [33,34]. Alien limb syndrome involves a dysfunction in neural circuits controlling body ownership and limb movement, resulting in distorted perceptions of the limbs and sensations of foreignness [35]. These examples highlight the intricate relationship between neurological factors and body image perception, shedding light on how changes in body schema and distortions in body image can manifest across various conditions.

### Applications of VR, AR, and MR in Clinical Rehabilitation

VR, AR, and MR not only provide new ways of conducting standardized, repetitive exercises and quantitative evaluations but also enable the development of novel therapeutic approaches [36]. Applications span disparate areas with different objectives and purposes. In relation to VR, these areas include stroke [37,38], Parkinson disease [39], Alzheimer disease [40], brain injury [41,42], unilateral spatial neglect [43], and pain management [44]. VR is also used in psychiatric disorders like specific phobias [45] and eating disorders [46,47]. Reviews suggest proposing XR-based tools [42,48].

In XR technologies, the boundary between reality and simulated reality is questioned [49]. The concept of the VR continuum, introduced by Milgram and Kishino in 1994 [50], maps out a range of environments stretching from the fully real to the fully virtual [51]. On one end, we find the physical world as we know it, and on the other end, we find a completely computer-generated environment, which is commonly called VR. Between these 2 extremes lies what is defined as MR, a middle ground that includes AR, where digital content is layered onto the real world, and augmented virtuality, where elements from the real world are brought into a mostly virtual setting. VR typically refers to interactive, computer-generated 3D environments that users can explore and manipulate in real time, usually through head-mounted displays (HMDs). These devices provide stereoscopic visuals and track both head and body movements, allowing the user’s perspective to shift naturally as they move. This creates a powerful sense of presence, as described by Jerald [52], and enables meaningful interaction with the virtual space. VR is now widely used across fields, such as entertainment, education, professional training, and health care [53]. While HMDs are currently the most common way to access VR, other immersive systems have also been developed. One notable example is the CAVE (Cave Automatic Virtual Environment), a room-sized installation with projected screens and motion tracking that allows multiple users to experience shared, walk-in virtual environments [54]. In contrast, Powerwall displays (large, high-resolution visualization screens) are typically associated with scientific visualization rather than immersive VR, mainly because they do not usually offer a deep sensorimotor immersion capacity. XR encompasses everything from AR and MR to fully immersive VR, acting as a “catch-all” for technologies that merge real and virtual environments in different ways. It is important to remember some basic aspects related to XR, which must be considered in rehabilitation applications. XR environments are typically characterized along the following key dimensions: immersion, presence, and embodiment [55]. These constructs, while distinct, are tightly interconnected and particularly relevant when examining the neurocognitive processes underlying motor rehabilitation. *Immersion* is the degree to which an XR system engages the user’s senses, excluding stimuli from the real world. The higher the level of immersion, the better the system can simulate a convincing sensory experience [56]. Immersion can be sensory (eg, high-quality graphics and 3D sound), interactive (ability to interact with the environment), and narrative (emotional engagement with the story or context of the virtual world). A VR headset with high resolution, directional audio, and a tactile controller increases immersion compared to a 3D world displayed on a screen (even a large screen) [57]. The concept of *multisensory experience* refers to the involvement of multiple senses (sight, hearing, touch, smell, and taste) to create a more realistic and engaging XR experience. Most XR experiences focus on sight and hearing, but adding tactile (haptics), thermal, or even olfactory stimuli can amplify the sense of presence and realism. While immersion refers to the degree of sensory stimulation provided by the XR system, its multisensory nature enhances the user’s engagement by stimulating multiple perceptual channels simultaneously. However, the sense of being in the virtual environment (presence) is also deeply intertwined with the sense of being the virtual body (embodiment) [58]. *Embodiment* is the sense of identification with a virtual body or avatar in the XR environment. This can be particularly useful in applications like rehabilitation, training, or neuroscience experiments. It can be influenced by the consistency between movements of the real body and those of the virtual body, avatar customization, and sensory feedback (eg, visual and tactile). This kind of view perspective can surely affect the embodiment level, but it is still not clear which one offers a better embodiment [59,60]. *Presence* issues refer to the subjective feeling of “being there” in the virtual environment, as if it were real. Presence is affected by immersion, and it occurs when the user’s brain accepts the virtual environment as a plausible reality, making the user forget that they are in a physical space. It can be affected by graphics quality, the realism of interactions, and latency. Moreover, it is associated with many other variables and is a constantly researched metric [5,59,61-63]. All the reported factors (multisensory experience, embodiment, and presence) are part of the concept of immersion.

*Cyber sickness* is a possible side effect of the use of XR technologies that must be considered, especially when used by subjects with disabilities. It arises from a conflict between sensory signals, such as when vision perceives movement in the virtual environment, but the body and inner ear remain static. Its symptoms are nausea, dizziness, headaches, visual fatigue, and disorientation. We can mitigate the risks of cyber sickness by improving frame rates, reducing latency, avoiding some particular visual movements, and designing more natural interactions (eg, allowing the user to “walk” physically instead of moving via controllers) [64]. Latency, often referred to as “motion-to-photon latency,” describes the delay between a user’s physical movement and the corresponding update in the XR environment [65]. When latency exceeds approximately 20‐30 milliseconds, it becomes perceivable and may cause sensory conflicts [35,48,49].

### Scope of the Review and Paper Organization

The research objectives include reviewing the literature on body representation and XR applications in patients with neurological conditions and focusing on technical aspects to propose innovative rehabilitation tools. An analysis of the literature found that in the last decades, several reviews concerning new technologies in neurorehabilitation have been published [66], with some studying the effects on multiple cognitive domains [67] or addressing particular conditions (eg, poststroke) [68]. On the other hand, the relevance of the body image concept and its potential to bring about significant changes in therapy have not yet been comprehensively reviewed. This introduces a gap in understanding the state of research regarding the use of novel adjunctive technology for beneficial intervention in the neuromotor domain and body image representation. To address this gap, we have conducted a scoping review aimed at providing current knowledge on VR, AR, and MR solutions for neurorehabilitation centered on the body image concept. Unlike previous reviews, which tended to focus on specific dimensions of body image disturbance in neurological rehabilitation, our work provides a comprehensive technological perspective. By detailing the XR devices, interaction modalities, and implementation settings used across studies, this review offers a practical resource for researchers aiming to explore or develop novel XR-based interventions. Our goal is to bridge the gap between clinical insight and technological implementation, enabling more informed decisions when selecting or designing XR solutions for body image rehabilitation in neurorehabilitation contexts.

This review aimed to (1) identify how VR, AR, and MR are used in motor neurorehabilitation contexts; (2) analyze how body representation is addressed; and (3) analyze the methodological approaches used in XR interventions. Regarding paper organization, the next section outlines the methods used, including the search strategy. Subsequently, the outcomes of the steps are presented, along with an analysis of various aspects of the research selected and retrieved. This is followed by a comprehensive discussion that reports the principal findings of the study based on an analysis of the results. Lastly, in the conclusion, we provide a summary of the work’s content and its relevance, and offer perspectives for future studies and insights.

## Methods

### Search Strategy

The PRISMA-ScR (Preferred Reporting Items for Systematic Reviews and Meta-Analyses extension for Scoping Reviews) guidelines were adopted [69]. The PRISMA-ScR checklist is provided in Checklist 1. The research protocol was registered by GD in the PROSPERO public registry (ID: CRD42023481092) before data extraction. Some changes were made to the registered research protocol. The objective was to critically analyze the existing literature on XR applications related to motor problems and somatic representation, which proposed new tools and experiments. This review was performed by searching 4 different electronic databases: PubMed, Embase, Scopus, and Web of Science. The search across the 4 databases was conducted on December 7, 2023, without any time limitations.

### Final Search Strategy Used in Each Database

For the development of the search strategy, several meetings were held with neurologist colleagues from the Spinal Cord Injuries Unit, Neuroscience Department, Pisa University Hospital, who are some of the authors of the research (CD and PB). The following groups of concepts were considered:

1. Target population: (Neurologic* OR Poststroke OR Post-stroke OR Post Stroke OR Stroke OR Brain Surgery OR Cerebral Palsy OR Paresis OR Spinal Cord Injury)
2. Body image and associated concepts: (Body Scheme* OR Body Imag* OR Body perception OR Dismorphism OR Bodily Self OR Bodily Self Consciousness OR Body Illusion* OR Body Matrix OR Body Model Theory OR Body Representation OR Body Swapping OR Embodiment OR Embodiment Cognition OR Ownership Illusion*)
3. Expositions and computer science concepts: (Virtual Reality OR Augmented Reality OR Mixed Reality OR 3D OR Tridimensional OR Avatar OR Virtual Embodiment OR Virtual Reality Reflection Therapy)
4. Experimental intervention outcome: (Neurorehabilitation OR Motor Recovery OR Motor Rehabilitation OR Action Understanding).

From these groups of concepts, the following updated query was developed: (Neurologic* OR Poststroke OR Post-stroke OR Post Stroke OR Stroke OR Brain Surgery OR Cerebral Palsy OR Paresis OR Spinal Cord Injury) AND (Body Scheme* OR Body Imag* OR Body perception OR Dismorphism OR Bodily Self OR Bodily Self Consciousness OR Body Illusion* OR Body Matrix OR Body Model Theory OR Body Representation OR Body Swapping OR Embodiment OR Embodiment Cognition OR Owner-ship Illusion*) AND (Virtual Reality OR Augmented Reality OR Mixed Reality OR 3D OR Tridimensional OR Avatar OR Virtual Embodiment OR Virtual Reality Reflection Therapy) AND (Neurorehabilitation OR Motor Recovery OR Motor Rehabilitation OR Action Understanding).

### Eligibility and Exclusion Criteria, Study Selection, and Data Extraction

Mendeley Reference Manager was used to identify and eliminate duplicate records within the library. After removing duplicates, 3 authors (OC, MM, and DM) independently evaluated titles and abstracts (n=98) based on the eligibility criteria. Articles were included if they involved XR interventions explicitly described as being for neurorehabilitation, reported user uptake data, reported implementation data, and were published in the peer-reviewed literature.

The articles selected by the 3 authors were independently read in full during the second screening phase to determine the final set of articles to be included in the review. In the case of conflicts, the 3 authors discussed the findings together, and if an agreement was not reached, the other authors provided the final judgment. The exclusion criteria were as follows: generic studies (ie, those addressing broad or nonspecific topics without a focused investigation into XR tools or original findings), editorials, reviews, studies that did not involve XR tools, and studies not available in English.

### Data Synthesis

A qualitative synthesis was conducted, in line with the PRISMA guidelines (PRISMA-ScR), to examine and compare methodological approaches across studies using XR for motor neurorehabilitation. All included studies were analyzed using a standardized data extraction form that captured study design, population characteristics, XR technology type, intervention protocol, outcome measures, and references to body representation (Multimedia Appendix 1). Studies were grouped by technology type and intervention context. When sufficient homogeneity in outcome measures (eg, motor performance scores and embodiment questionnaires) was present, basic quantitative aggregation (eg, frequency of use and effect direction) was performed descriptively. However, no formal meta-analysis was conducted due to high variability in study design, outcome measures, and intervention protocols.

### Study Quality

To assess the quality of each study, the Newcastle-Ottawa Scale (NOS) was used. The NOS is a collaboration between the University of Newcastle, Australia and the University of Ottawa, Canada. It has been developed to assess the quality of nonrandomized studies. A “star system” is used, and a study is judged on 3 perspectives: the selection of the study groups, the comparability of the groups, and the ascertainment of either the exposure or the outcome of interest for case-control or cohort studies [70]. Each study was assigned a score of up to 9 stars, except for cross-sectional studies, which could receive a maximum of 8 stars.

For case report studies, we used a dedicated scale published by Murad et al [71], which assigns a maximum of 8 points to each study. Case reports and case series are uncontrolled study designs, which are known to have an increased risk of bias. This guide provides a framework for evaluating the methodological quality of case reports and case series based on the domains of selection, ascertainment, causality, and reporting, and provides signaling questions to aid evidence-based practitioners and systematic reviewers in their assessment. The tool for evaluating the methodological quality of case reports and case series is based on leading explanatory questions concerning the following aspects of a clinical trial: (1) Selection (Does the patient(s) represent(s) the whole experience of the investigator (center) or is the selection method unclear to the extent that other patients with similar presentation may not have been reported?); (2) Ascertainment (Was the exposure adequately ascertained? Was the outcome adequately ascertained?); (3) Causality (Were other alternative causes that may explain the observation ruled out? Was there a challenge/rechallenge phenomenon? Was there a dose-response effect? Was follow-up long enough for outcomes to occur?); and (4) Reporting (Is the case(s) described with sufficient details to allow other investigators to replicate the research or to allow practitioners to make inferences related to their own practice?) [71].

These assessments were integrated into the synthesis to confirm the strength and reliability of the findings, and the scores and characteristics of the studies are reported in Multimedia Appendix 1.

## Results

### Search Results and Study Characteristics

**Figure 1.**
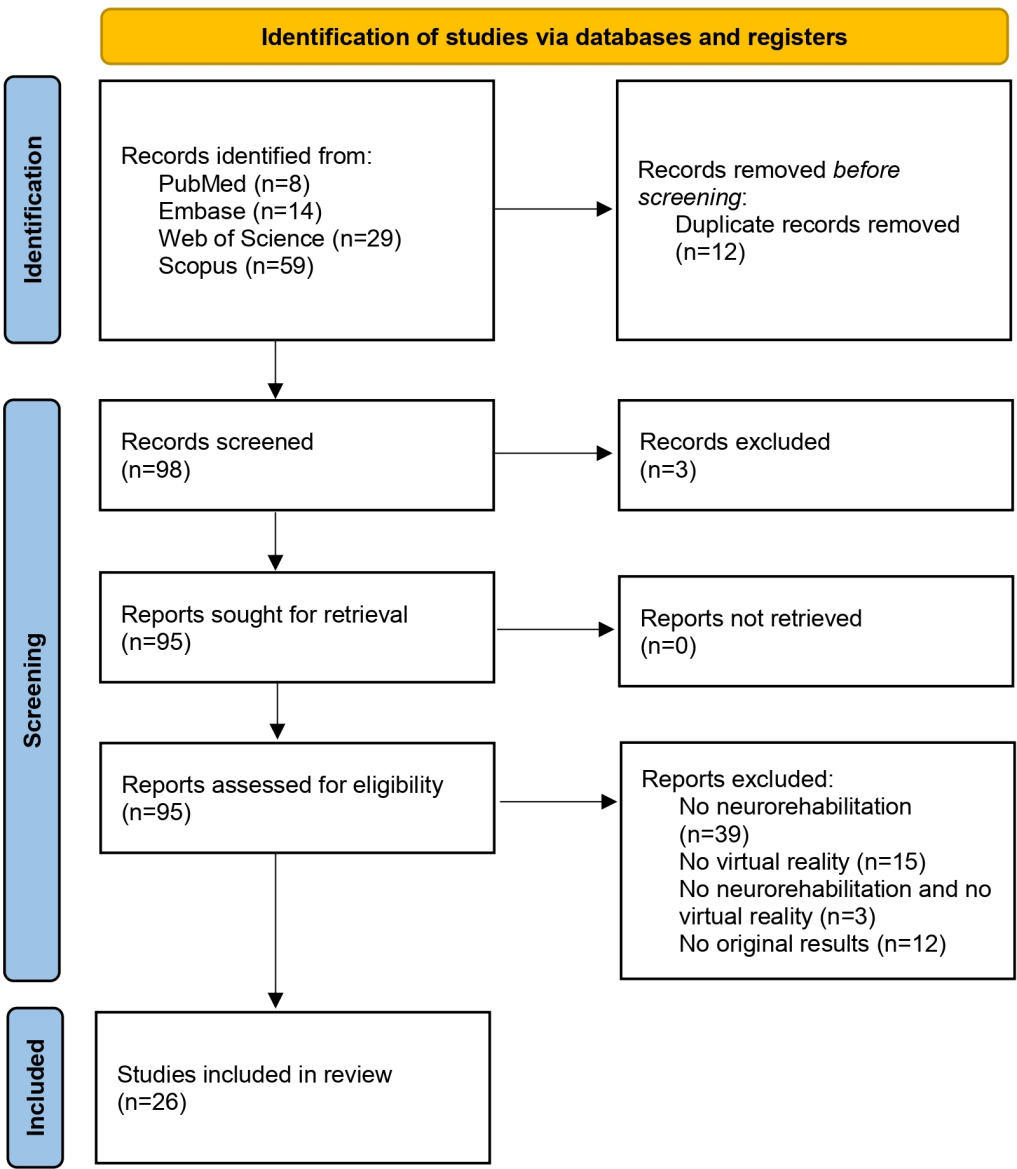
PRISMA (Preferred Reporting Items for Systematic Reviews and Meta-Analyses) 2020 flow diagram of article selection.

From PubMed, 87 results were initially obtained. After applying the electronic database filter for only those articles that had the keywords in their titles and abstracts, 8 articles were identified. Using the Embase search engine, we obtained 14 articles. Querying the Scopus search engine with the search string, we identified 581 records (“all field” search). The filter “title and abstract” was then selected, and the search was repeated by using “article” and “computer science” as filters. At this point, 59 articles from Scopus were identified. Using the Clarivate Web of Science archive, we identified 29 records. The PRISMA flow diagram is presented in Figure 1.

Out of the total 110 records obtained from the 4 search engines, 12 duplicate records were removed, resulting in 98 records. Three records were excluded as they were articles related to proceedings, and the remaining records were scientific papers, either experimental or based on systematic or narrative literature reviews (Figure 1). After screening the full text and applying the exclusion criteria, 26 articles were included in the review. By the end of the identification process, 76% (84/110) of articles in the initial set were excluded.

### Geographical and Timeline Distribution

The articles included in this review involved studies carried out in Italy (n=6), Switzerland (n=5), Japan (n=3), China (n=2), Portugal (n=2), United Kingdom (n=1), Spain (n=2), United States (n=1), Mexico (n=1), New Zealand (n=1), Korea (n=1), and Brazil (n=1). Table 1 presents the geographical and timeline distributions of the studies. Research has increased in the last 5 years, with over 80% (21/26, 81%) of studies being published from 2019 to 2023 (Table 1).

**Table 1.** Geographical and timeline distribution of the studies included in the review (N=26).

Country	Year									Total per country
	2013 (n=1)	2016 (n=1)	2017 (n=2)	2018 (n=1)	2019 (n=2)	2020 (n=3)	2021 (n=4)	2022 (n=5)	2023 (n=7)	
Italy	0	0	0	1	0	1	1	1	2	6
Switzerland	0	0	1	0	0	0	1	2	1	5
Japan	0	0	1	0	1	0	1	0	0	3
China	0	0	0	0	0	0	0	0	2	2
Portugal	0	0	0	0	0	0	0	1	1	2
United Kingdom	0	0	0	0	0	0	0	0	1	1
Spain	1	0	0	0	1	0	0	0	0	2
United States	0	0	0	0	0	0	0	1	0	1
Mexico	0	0	0	0	0	0	1	0	0	1
New Zealand	0	0	0	0	0	1	0	0	0	1
Korea	0	0	0	0	0	1	0	0	0	1
Brazil	0	1	0	0	0	0	0	0	0	1

### Study Design and Population

Among the 26 studies analyzed, variability was observed in the sample size of participants and in the type of participants involved in the analysis. One study included a single patient [72], and another study involved an analysis of 1 patient and 5 healthy male control participants [73]. The largest sample (113 healthy participants and 16 patients who had experienced stroke) was noted in the study by Dong et al [74]. The upper limbs and hands were the most frequently evaluated body parts (n=19), and 3 studies assessed the application of VR to the entire body. Two studies involved children. The study by Phelan et al [75] included 3 boys and 5 girls with upper limb motor impairment (mean age of 13 years), and the study by Garcia-Hernandez et al [76] included 19 children with cerebral palsy (mean age of 8 years). Of the 26 studies, 12 included only healthy participants, 11 included only patients, and 3 included both healthy participants and patients. Only 2 articles involved studies that could be considered to have a case-control design. The main pathological conditions identified were poststroke status (n=6) and spinal trauma (n=2).

### Intervention Evaluation Studies and Outcome Measures

Among the selected studies, 16 explicitly stated that they involved body perception. Most studies (n=14) investigated the embodiment of virtual representations of the limbs, and in particular, 6 studies focused on the sense of agency toward virtual limbs. Matsumiya [77] explicitly considered motor control.

Administration of questionnaires represented the most used method for evaluating the usefulness of the proposed systems (18 out of 26 studies). Three studies indicated the use of standard assessment systems or those derived from standards, such as the Box and Block test. Caola et al [78] explored whether it is possible to induce a sense of body ownership over a virtual body part during visuomotor inconsistencies, with or without the aid of concomitant visuotactile stimulations. The study enrolled 45 healthy participants. A small vibrator was placed in the middle of the dorsal part of the participant’s right hand and controlled via an Arduino board. From a first-person perspective (FPP), the participant watched a virtual tube moving or an avatar’s arm moving, with or without concomitant synchronous visuotactile stimulations on the hand. Three different virtual arm or tube speeds were investigated while all participants kept their real arms still. The primary result from this research was that it is feasible to generate a feeling of ownership over a virtual body part, even in the presence of a significant discrepancy between the real and virtual limbs’ visual and motor aspects [78], and the findings were derived from a questionnaire-based approach [79]. Some studies indicated the extraction of indices from physiological data, such as electroencephalography (EEG; n=12), electromyography (EMG; n=2), magnetic resonance imaging (MRI; n=1), galvanic skin response (n=1), electrodermal activity (n=1), and motor-evoked potential data (n=1). The manual controllers provided with VR systems can easily be used to detect movement characteristics, with 4 studies using indices derived from their data. Gaze analysis was not widely used among the selected studies, appearing in only 2 studies. In an experimental study, Kaneko et al [80] clarified the effect of kinesthetic perception illusion induced by visual stimulation (KINVIS) on upper limb motor function and the relationship between motor function and resting-state brain networks. Eleven patients with severe upper limb paralysis were enrolled, and functional MRI was used to evaluate motor function and resting-state brain functional connectivity. The cognitive phenomenon of KINVIS was described as the feeling of one’s body moving during sensory input, even though the body is actually in a resting state. The approach involved a FPP and an AR system with monitors. The participants received visual stimulation for 20 minutes together with neuromuscular electrical stimulation. The patients were seated on a chair with their forearms on a table. The hand movement of the unaffected side was recorded before the intervention. The movement task involved hand opening and closing. This task was executed using the unaffected side, and the recording was flipped to reflect the movement of the affected side. The results of motor function (Fugle-Meyer Assessment) and spasticity (Modified Ashworth Scale) showed significant improvement following the intervention. Assessment and evaluation were carried out using the Action Research Arm Test, the Box and Block Test, the Motor Activity Log, and MRI data analysis [80]. The selected studies were quite heterogeneous, and thus, there were differences in the types of evaluation tools. However, given the extensive use of self-report tools, such as questionnaires [81], there is still no clarity regarding the objective and standardized evaluation of the effectiveness of XR use. Inamura et al [82] determined the feasibility of a VR system (whether it has enough effect on the sense of agency and sense of ownership) in healthy participants before conducting experiments in patients with phantom limbs. The Kinect V2 VR system was used. In this system, a virtual avatar performs a motion identical to that of the participant using a motion-capture device. The participant wore a 3D HMD to experience seeing through the eyes of the avatar. Six conditions of avatar representation were used: 2 arms (normal human arm and robot arm) and 3 lengths of each arm (short, medium, and long). The participant executed elbow flexion-extension movements of the right arm, which caused the same movements in the VR avatar’s arm. The findings indicated that the perceived length of the arm was altered based on the displayed arm’s length within the VR setting. Through analysis of questionnaire responses, it was determined that there was no adverse impact on the sense of agency. Additionally, it was observed that the sense of ownership was stronger when participants viewed a normal human arm than when they viewed a robot arm. The evaluation was carried out using a questionnaire and gesture data analysis. After the induction movement, the subjective sense of the length of the right arm was measured by a pointing gesture of the left hand [82]. Pozeg et al [83] investigated changes in body ownership and chronic neuropathic pain in patients with SCI using multisensory own body illusions and VR. Their study enrolled 20 patients with SCI and paraplegia and 20 healthy control participants. The HMD used showed a real-time (or delayed) video of dummy legs from a distance and angle that corresponded to the participant’s first-person viewpoint. The findings revealed that individuals with SCI had reduced sensitivity to multisensory stimuli that generated an illusion of leg ownership in comparison to those without such injuries (healthy controls). Furthermore, the sense of leg ownership diminished over time following SCI. Interestingly, there were no discernible distinctions between the groups in terms of overall body ownership, as assessed by a full-body illusion (FBI). The assessment and evaluation methods were as follows: the virtual leg illusion was assessed with a 9-item questionnaire adapted from body illusion studies, the FBI was assessed with a 7-item questionnaire, and actual neuropathic pain was assessed with a visual analog scale [83]. The VR, AR, and MR tools were mainly used to improve sensorimotor function in adults with neurological disorders and to test the feasibility of the devices. While body representation may be activated during the use of these tools, of the 26 included studies, 6 had an outcome measure on embodiment, 5 had an outcome measure on ownership, and 2 had an outcome measure on self-location perception.

### Quality Assessment

In the quality assessment, 13 studies obtained the maximum score, and the remaining studies scored slightly below the maximum score. However, in 1 study, the results were not adequately reported [84]. The main reason for not obtaining the maximum score was related to the limited sample size and inadequate reporting of the results. However, it is important to emphasize that a large proportion of the studies considered were feasibility or usability studies.

### XR Technologies and Technical Details of the Hardware and Software

In the last 2 decades, there has been a shift from complex and bulky systems that require high-performance computers to more affordable and agile alternatives. The emergence of budget-friendly XR devices, such as Oculus Rift and HTC Vive, has undeniably propelled research involving the use of VR technologies. Among the examined studies, 7 used Oculus Rift (Dk1 and Dk2), 3 used the more recent and portable Oculus Quest 1 and 2, 6 used HTC Vive, 1 used other devices (typically older and more expensive), 1 used Valve Index, and 6 did not specify the system used. Two studies used standard, large monitors instead of HMDs, and although they were not immersive, the modality of the experiment shared many similarities with the others. Thus, they were included in the study list. Camardella et al [85] showed the design, implementation, and first evaluation of a gaming scenario for the upper limb rehabilitation of children with cerebral palsy, using one of these recent devices. The experiment was developed with 8 healthy participants (upper limb), and it involved the use of VR in the FPP with Oculus Quest 2. In this case, the VR environment depicted a magical training ground for wizards, surrounded by mountains and trees, with a large rune on the floor marking the spell-casting area. Players used their index finger as a wand to draw symbols in the air, casting spells at enemies. The accuracy of symbol drawing determined spell potency, with reference to a 2D sample provided. Two custom lightweight haptic thimbles provided tactile feedback. The assessed conditions included speed reference presence and feedback type (haptic or visual) related to tracking velocity. Preliminary examinations conducted on these healthy participants revealed that the introduction of haptic feedback did not notably change the perception of absolute speed or the capability to uphold a steady self-selected reference speed. Nevertheless, when participants were directed to adhere to a predetermined reference speed, the incorporation of haptic feedback improved performance by enhancing smoothness and diminishing speed-tracking errors. However, it is worth noting that only smoothness demonstrated a statistically significant improvement. The evaluation was carried on through the analysis of a dataset built using the recorded hand speed as a feature [85]. In general, these devices use hand trackers for navigation within the virtual space. However, only 5 studies explicitly mentioned the use of hand trackers [62,73,86-88]. Fregna et al [86] chose to use the optical-based hand tracking system embedded in Oculus Quest 2. In 3 studies, the authors reported using Microsoft Kinect for interaction with the virtual scene [5,76,82]. This confirms that this device, which was originally created for video games and had very little success in its domain, found new life in the research field worldwide. Heinrich et al [87] reported the use of the Leap Motion device, which is smaller than the Kinect device. Llobera et al [73] opted for an InterSense 6-degree wand device. In VR systems, users can experience either a first-person or third-person view of the synthetic world around them (where they see their avatar). Most of the identified studies (n=24) exclusively used the FPP, which is also referred to as the first-person view. Tambone et al [89] and Borrego et al [5] conducted a comparison between the FPP and first-person view, noting a higher level of embodiment in the first mode. Song et al [90] induced a third-person perspective full-body illusion (TPP-FBI) with VR in 19 patients with stroke. The study used the TPP Valve Index VR headset plus controllers in the following 4 experimental conditions: synchronous visual-tactile stimulation on the back, synchronous visual-tactile stimulation on the arm, nonsynchronous visual-tactile stimulation on the back, and nonsynchronous visual-tactile stimulation on the arm. The experimenter randomly touched the participant’s back or arm with a physical bar. In synchronous conditions, virtual and physical bar stimulation matched temporally and spatially. The findings demonstrated that VR could trigger the sensation of ownership of a TPP-FBI in patients with stroke, akin to how it does in healthy individuals, through synchronous visual-tactile stimulation of a specific body part (the back or upper limb). Furthermore, it was observed that stimulating the back could evoke a more pronounced sense of a TPP-FBI compared to stimulating the affected upper limb. This suggests that for patients with stroke experiencing limb dysfunction, stimulating the back may be more effective in inducing a robust sense of ownership from a TPP. In this study, questionnaire scores reflected the subjective experience of the participants, and self-location drift values reflected the objective self-location perception [90]. To provide feedback that is crucial for creating an illusion, Pais-Vieira et al [72] used a custom-developed thermal tactile sleeve. Moreover, Shokur et al [91] adopted a tactile t-shirt equipped with eccentric mass vibrators, and Matsumiya et al [77] used a Phantom Force feedback device. The remaining studies did not specify the use of special feedback devices. Five studies incorporated EEG for recording brain responses during experiments: Pais-Vieira et al [72] and Batista et al [92] used a Brain Product GmbH EEG system, Lim et al [93] used a Wearable Sensing device, and Llobera et al [73] and Sanford et al [94] used a g.USBAmp system for recording both EEG and EMG. Two studies used transcranial stimulators for different purposes: Buetler et al [88] used them for detecting motor responses, and Lim et al [93] used them for enhancing a virtual hand illusion. Some studies used other kinds of devices for the experiments: Wenk et al [62] and Matsumiya et al [77] used an eye tracker, and Tambone et al [89] used an additional OLED display.

Most studies did not specify the 3D engine used. However, 8 studies explicitly claimed to have used Unity 3D [5,62,74,76,84,85,89,92], and only 1 study mentioned the use of Unreal Engine [75]. In the areas of the research and development of XR applications for rehabilitation, Unity 3D stands out as the preferred platform over Unreal Engine for several reasons. First, Unity is widely regarded for its accessibility and ease of use. Its gradual learning curve and extensive documentation make it particularly appealing to developers and researchers, even those without a strong background in gaming or advanced graphics. The scripting process in Unity, which is based on C#, is simpler compared to Unreal’s reliance on C++, further lowering the barrier to entry. Additionally, Unity’s flexibility across mobile devices and AR platforms is a major advantage. It offers native support for a wide range of AR devices, including HoloLens, Magic Leap, and various mobile headsets, through seamless integration with tools like ARKit and ARCore. This streamlines development for projects that aim to reach a diverse array of compatible devices, ensuring greater accessibility for end users. Unity also benefits from its strong presence in academic and research communities. Its ecosystem includes a wealth of third-party plugins and libraries tailored for scientific and rehabilitation applications, such as Vuforia, MRTK for HoloLens, and other medical AR solutions. These resources make it easier to rapidly prototype rehabilitation systems and integrate advanced tools for motion analysis and interaction. Finally, cost and licensing considerations play significant roles. While Unreal excels in delivering high-quality graphics for video games and advanced media, Unity is often more budget-friendly for small research groups or projects that do not prioritize cutting-edge visual fidelity. This affordability makes it an attractive option for academic and clinical research settings.

As for other additional software, MakeHuman, which was cited by Wenk et al [63] and Odermatt et al [95], is widely used for avatar creation in XR applications. Multimedia Appendix 1 summarizes the main characteristics of the studies and the XR tools included in this review in the order of the year of publication.

## Discussion

### Summary of Evidence

Following a meticulous screening process, 24% (26/110) of studies met the inclusion criteria, signifying a reasonable interest in this area. Notably, over 80% (21/26, 81%) of the studies were published within the past 5 years, indicating a recent surge in research attention.

Geographically, the studies were predominantly conducted in Italy, Switzerland, and Japan. They covered a diverse array of neurological conditions, including poststroke rehabilitation, spinal trauma, cerebral palsy, and others. Notable contributors to this body of research include studies by Phelan et al [75], Garcia-Hernandez et al [76], Tambone et al [89], Borrego et al [5], Pais-Vieira et al [72], Shokur et al [91], and Matsumiya et al [77].

Interestingly, the assessment methods used varied widely across studies. Quality assessment revealed that the limitations of the included studies primarily stemmed from issues such as small sample sizes and inadequate reporting of results. It is worth noting that a significant portion of the included studies focused on feasibility and usability rather than strictly efficacy.

There is an ongoing debate in motor rehabilitation using XR regarding the optimal perspective (FPP or TPP). Current research suggests that each perspective offers distinct advantages depending on the context and desired outcome [5,83,89]. Studies have shown that the FPP generally enhances the sense of embodiment and self-presence, as it aligns the user’s visual perspective with their actions, fostering a stronger connection to the virtual avatar [5,83,86,88,95]. This may be beneficial for tasks requiring fine motor skill retraining or when the goal is to replicate natural movements. However, some studies have suggested that the TPP can also provide a comparable level of embodiment if the user identifies closely with the avatar [61]. The TPP may offer advantages for tasks requiring spatial awareness or understanding the body’s overall posture and movement. For example, observing an avatar from behind can provide clearer feedback on errors in form during rehabilitation exercises. This can be particularly useful in therapies targeting balance or coordination. The effectiveness of the FPP versus the TPP often depends on the specific rehabilitation task and individual needs. For instance, patients recovering from conditions that impair body awareness might benefit from the external feedback provided by the TPP, while others might achieve better outcomes with the immersive nature of the FPP. Given the mixed findings, a hybrid approach combining both perspectives or tailoring the choice based on the user’s progress and preferences might be the most effective strategy. Borrego et al [5] compared the experience of embodiment and the sense of presence in patients who have experienced stroke and healthy subjects, using both the FPP and TPP. The results indicated that the FPP enhances the sense of embodiment, while the TPP improves the awareness of global movement.

For feedback, some studies used custom devices like thermal tactile sleeves, tactile t-shirts, and force feedback devices [72,77,89,91]. Five studies recorded brain responses using EEG, while others used transcranial stimulators, eye trackers, and additional displays [72,88,89,92-94]. Haptic feedback plays a crucial role in XR systems for motor rehabilitation by enhancing the user’s ability to understand and control their movements through tactile and force-based signals. This interaction not only helps patients correct their motor patterns in real time but also boosts their motivation, as the tactile sensations make rehabilitation more engaging and rewarding. Moreover, haptic devices enable the realistic simulation of object manipulation, providing a safe environment to practice practical and fine motor skills. By integrating tactile feedback with visual and auditory stimuli, haptic systems create a cohesive multisensory experience that strengthens the user’s sense of presence and embodiment within the virtual environment. This sensory coherence fosters the illusion that the user’s virtual body is an extension of their physical self, which is crucial for stimulating brain plasticity and supporting motor recovery. While the implementation of haptic feedback has some challenges, such as cost, complexity, and the need for precise calibration, its ability to deliver immersive and effective therapy makes it a valuable component of XR-based rehabilitation systems. Bortone et al [96,97] showed that haptics can enhance the involvement and engagement of patients, provide congruent multisensory afferent feedback during motor exercises, and benefit from the flexibility of XR in adapting exercises to the patient’s needs. Rätz et al [98] developed and tested a rehabilitation device for the upper limbs that uses haptic feedback across the entire hand. Patients with a history of stroke and therapists who tested the device appreciated its ability to provide realistic haptic interaction, which is beneficial for recovering motor skills and enhancing the transfer of skills acquired to daily activities.

### Comparison to Prior Work

The body of literature on the use of XR technologies in neurorehabilitation reflects a diverse array of findings and perspectives, and this scoping review sheds light on both the promises and challenges of integrating this innovative technology into clinical practice [42,48].

Ventura et al [84] conducted a systematic review that underscored the potential of body ownership illusion through VR in limb rehabilitation after stroke, highlighting its role in accelerating motor recovery by fostering a sense of embodiment [99]. This finding resonates with embodied cognition theories, which emphasize the reciprocal relationship between bodily states and cognitive functions, and this is particularly evident in language processing among individuals with neurological disorders [100]. Leeb and Pérez-Marcos [101] further explored the synergies between brain-computer interfaces and VR, highlighting their transformative potential in redefining neurorehabilitation paradigms by integrating motor-cognitive training with evidence-based neuroscience principles. In a meta-analysis, Maier et al [102] delved into the comparative efficacy of specific VR and nonspecific VR systems for upper limb rehabilitation after stroke, revealing that specific VR systems tailored explicitly for rehabilitation yielded superior outcomes compared to nonspecific VR systems [102]. These findings underscore the importance of designing VR interventions that are specifically tailored to address the unique rehabilitation needs of patients with neurological conditions. Moreover, immersive VR environments have emerged as promising tools for alleviating pain and modulating body perception, offering novel therapeutic avenues for managing conditions characterized by distorted body image [103,104]. Perez-Marcos et al [105] emphasized the role of VR in empowering patients through engaging and motivating training approaches, integrating motor-cognitive training with evidence-based principles to foster self-management and ownership of the rehabilitation process. While the potential of VR in neurorehabilitation is vast, Tieri et al [49] cautioned against overstating its superiority over conventional rehabilitation techniques, highlighting the need for a nuanced understanding of its efficacy across diverse patient populations and clinical contexts. Similarly, Dieguez and Lopez [106] underscored the importance of clinical reporting in comprehensively understanding abnormal body representations in neurological disorders, paving the way for targeted interventions informed by empirical evidence. Furthermore, Pulay [107] proposed leveraging eye-tracking and EMG devices in VR to facilitate cognitive and motor development in children with severe physical disabilities, offering a glimpse into the transformative potential of technology-assisted interventions for enhancing rehabilitation outcomes. In parallel, Christ and Reiner [108] explored the theoretical underpinnings and clinical applications of rubber hand and virtual hand illusions, offering insights into their implications for rehabilitation. Additionally, Hesse et al [109] traced the evolution of robotic devices for motor rehabilitation, highlighting their role in delivering intensive and task-specific therapy approaches for stroke and spinal cord injuries and thereby opening new vistas for optimizing motor recovery outcomes.

Williamson et al [1] investigated the psychological impact of XR interventions in SCI rehabilitation. Their findings suggest that XR-based therapies offer psychological benefits, including enjoyment, relaxation, and positive distraction. The review concluded that immersive interventions show potential for supporting psychological well-being during SCI rehabilitation. Lu et al [110] evaluated the integration of 3D and 4D digital human modeling with XR across 16 studies addressing conditions such as neglect, anorexia nervosa, bulimia nervosa, and type 2 diabetes. The results indicated improvements in functional, physical, psychological, and overall health outcomes. Nonetheless, the authors emphasized the need for larger sample sizes, longer-term follow-up, and standardized outcome measures to better assess the reliability and efficacy of these interventions. Previous reviews explored the use of XR in neurological disease management and psychological rehabilitation, emphasizing its role in the treatment of chronic conditions and the improvement of psychological health. Some authors [111] explored how XR technologies could reshape the treatment of neurological diseases, proposing that XR may offer innovative pathways for patient care and rehabilitation. The article highlighted the potential of XR to revolutionize treatment approaches for neurological diseases. Chenais and Görgen [112] provided an overview of current uses and future directions of immersive interfaces in clinical settings. Their review highlighted how these technologies might enhance both patient engagement and therapeutic outcomes while calling for further investigation to optimize their clinical utility. Previous literature reviews have focused on the application of XR in neurorehabilitation and highlighted its potential, but have noted the need for further efficacy research. Schuermans et al [113] conducted a systematic review on XR-supported rehabilitation and injury prevention in musculoskeletal contexts, finding added value in both sports injury prevention and recovery. Figeys et al [114] assessed MR interventions in acquired brain injury rehabilitation, noting encouraging outcomes in areas such as upper limb motor function, gait, cognition, and lower-limb mobility. However, they also pointed out the early-stage nature of much of the research, with many studies still at the prototype stage. Taghian et al [115] examined VR/AR applications in biomedical engineering, highlighting their use in fields such as rehabilitation. Their review emphasized the transformative potential of VR/AR technologies while stressing the importance of continued research to unlock their full benefits.

Compared with previous work, this scoping review focused more on technical aspects to propose innovative rehabilitation tools. Overall, the review underscores the burgeoning interest and promising potential of XR in neurorehabilitation while also advocating for more standardized evaluation methods to ensure robustness and comparability across studies.

### Strengths and Limitations

This review captured a broad range of populations and health care providers across several countries. To ensure comprehensive coverage, multiple databases were searched without time restrictions. Despite the methodological rigor applied, several limitations should be acknowledged. Important differences were observed among the studies reviewed, including variations in sample size, duration of follow-up, treatment protocol, and study design. Variability in the sample population, particularly in terms of demographics and pathology, was a significant limitation. Moreover, there were limitations related to heterogeneity in treatments that resulted from variations in methodology, duration, and adherence to standard protocols, which consequently introduced uncertainty in the analysis. The absence of comparison or control groups in some studies might have affected the validity of the results. Moreover, the research had a wide scope. It was not possible to conduct a comprehensive outcome analysis because of the heterogeneity of the included research. There should be more specific and focused research questions geared toward evaluating outcomes, more evidence, and more consistent measures, and a systematic review and meta-analysis would be a valuable addition to the literature. The purpose of this literature review was to provide a broad overview of studies that adopted XR in the field of functional and cognitive neurorehabilitation, using the concept of body representation in its various forms.

While the review encompassed a comprehensive search across major electronic databases, the exclusion of other databases and gray literature may have overlooked relevant studies. Moreover, limiting the analysis to English-language articles potentially excluded methodologically robust studies published in other languages, leading to information loss.

### Conclusions

This review enabled a critical evaluation of evidence and identification of knowledge gaps [116]. Through repetitive, task-specific training and reinforcement learning mechanisms, XR interventions deliver personalized and engaging rehabilitation approaches [116]. The use of animated avatars further supports motor learning via mirror neuron activation [117]. The degree of immersion, determined by sensory and motor engagement, shapes a user’s sense of presence, bodily ownership, and agency within the virtual space [49,118], fostering deeper involvement in rehabilitation tasks and potentially improving motor and cognitive outcomes [49].

Nevertheless, several limitations remain. Few reviewed studies addressed cybersickness, and only a minority examined the integration of AR technologies, which may partially mitigate such effects [60]. AR offers unique advantages for poststroke rehabilitation by leveraging familiar environmental cues and facilitating the transfer of acquired skills to daily activities. Integrating virtual elements into real-world settings enhances embodiment, motor control, and coordination while maintaining the natural context of rehabilitation exercises and promoting engagement [63].

Recent research highlights the potential of “AR pass-through” modes available in new-generation headsets (eg, Oculus Quest 3 and Apple Vision Pro), which allow patients to perceive their real environment while interacting with virtual elements [98,114]. This continuity between real and virtual contexts reduces spatial disorientation and cybersickness, improving safety and comfort during rehabilitation. Moreover, AR enables direct interaction with real objects, eliminating the need for complex haptic feedback and simplifying system design without compromising functionality [119,120].

Overall, AR tends to induce less cybersickness than fully immersive VR because it preserves visual access to the real environment and reduces sensory conflicts. However, factors, such as headset weight, rendering latency, and intensive movement tasks, may still contribute to discomfort if not properly managed.

In conclusion, XR technologies, particularly AR and MR, show strong potential to enhance patient engagement and support personalized, multisensory therapeutic approaches that foster neuroplasticity and adherence to rehabilitation protocols.

### Future Research and Application

Future research should analyze the synergistic effects of VR and AR interventions and their impacts on motor recovery and quality of life in clinical populations. Additionally, efforts to address the limitations of existing studies and promote standardized reporting practices will contribute to advancing the field of VR-based neurorehabilitation.

Emphasizing the practical implications for clinicians will help bridge the gap between research and application, ensuring that these innovations are used effectively in real-world settings. Future studies should focus on long-term outcomes associated with XR interventions, as well as their effects on various patient populations. The success of XR interventions may depend significantly on patient engagement and individual preferences.

## Supplementary material

10.2196/63487 Multimedia Appendix 1 Relevant literature involving virtual, augmented, and mixed reality applications for motor neurorehabilitation focused on body representation.

10.2196/63487 Checklist 1 PRISMA-ScR checklist

## Abbreviations

AR: augmented reality
EEG: electroencephalography
EMG: electromyography
FBI: full-body illusion
FPP: first-person perspective
HMD: head-mounted display
KINVIS: kinesthetic perception illusion induced by visual stimulation
MR: mixed reality
MRI: magnetic resonance imaging
NOS: Newcastle-Ottawa Scale
PRISMA-ScR: Preferred Reporting Items for Systematic Reviews and Meta-Analyses extension for Scoping Reviews
SCI: spinal cord injury
TPP: third-person perspective
VR: virtual reality
XR: extended reality
