# Supplementary material for: Virtual, Augmented, and Mixed Reality for Motor Neurorehabilitation: Scoping Review Focused on the Role of Body Representation
Source: JMIR XR Spat Comput. 2025 Dec 17;2:e63487. doi: 10.2196/63487 (PMC13202512; doi:10.2196/63487)
Supplement: Multimedia Appendix 1 [file xr-v2-e63487-s001.docx]

**Multimedia Appendix 1:** Relevant literature involving virtual, augmented, and mixed reality applications for motor neurorehabilitation focused on body representation.

| **First author, institution, title, journal** | **Year** | **Objective** | **Population and condition** | **Body part,**  **XR type,**  **perspective** | **VR, hardware, and software (HW/SW), tools, and View** | **Experimentation details** | **Results** | **Assessment**  **/Evaluation methods** | **Quality score*** |
| --- | --- | --- | --- | --- | --- | --- | --- | --- | --- |
| Dong, Y. [1]  Beihang Univ, Beijing Adv Innovat Ctr Biomed Engn, Sch Biol Sci & Med Engn, Key Lab Biomech & Mechanobiol, Minist Educ, Beijing  *A haptic-feedback virtual reality system to improve the Box and Block Test (BBT) for upper extremity motor function assessment*  Virtual Reality | 2023 | To test a haptic-feedback, virtual reality-based version of the Box and Block Test. | 113 healthy and 16 post-stroke subjects | Upper Arm, VR, FPP | Oculus Rift, Chai3D | The VBBT (virtual BBT) task required users to move virtual blocks as many as possible from one compartment  of a virtual box to the other within one minute. During the task, a haptic device was used to collect data, including the trajectory, velocity, and grasping force | Quantitative performance in the VBBT showed improved reliability  compared to that of the BBT, although subjects' performance in the VBBT presented a stronger age-related correlation than that of BBT. | Assessment: with Mini-Mental State Examination (MMSE), Brunnstrom Stage, FMA-UE, ARAT and BBT.  Evaluation:  - intrinsic motivation inventory (IMI)  - questionnaire  - Haptic device data analyses. | 8/9 |
| Batista, D. [2]  Institute for Systems and Robotics - Lisboa, Instituto Superior Técnico, Universidade de Lisboa, Lisbon, Portugal  *Effect of head-mounted virtual reality and*  *vibrotactile feedback in ERD during motor imagery*  *Brain-computer interface training*  BRAIN-COMPUTER INTERFACES | 2023 | Testing motor Imagery-BCI (MI-BCI) setup for evoking stronger sensorimotor rhythms in VR | 19 healthy subjects | Upper Arm, VR, FPP | Oculus Rift CV1.  EEG by LiveAmp; Brain Products GmbH.  Oculus Rift hand controllers. | A set of 6 conditions were used (various combinations of MI and VR/no VR). Two of them (non-VR and non-embodied abstract feedback) are used as controls.  Two conditions  involved vibrotactile stimulation. | Through the acquisition of EEG signals and analysis of Alpha and Beta ERD and the utilization of a virtual environment, NeuRow, along with haptic feedback implemented as vibrotactile stimulation in this research, resulted in significantly enhanced contralateral ERD. These enhancements were comparable to those observed during actual motor execution. However, the use of VR HMD alone did not yield comparable outcomes, akin to merely utilizing a computer monitor without haptic feedback. | Evaluation:  EEG Data Analysis | 7/8 |
| Wenk, N. [3]  University of Bern  *Effect of immersive visualization technologies on cognitive load,*  *motivation, usability, and embodiment*  Virtual Reality | 2023 | The goal of this study was to evaluate the potential benefits of more immersive technologies using head-mounted displays (HMD), compared to a standard 2D screen. | 20 healthy subjects  (15 females, five males) | Upper Arm, AR and VR, FPP | HTC Vive, Meta 2 AR. Unity 3D. MakeHuman | This study compares three modalities: Immersive VR, AR, and a 2D screen. In each modality, participants performed the same dual-task visualized with different displays. The motor task consisted of sequentially reaching 120 fruits that appeared randomly in one of 22 possible locations. After performing the dual motor-cognitive task with each modality, participants were requested to fill in questionnaires to report their subjective cognitive load, motivation, technology’s usability, and embodiment. | The average subjective cognitive load (RTLX) was lower with IVR compared to other modalities, but the differences did not reach statistical significance. However, the authors observed a significant effect of the modality on the physical demand subscale of the RTLX. Interest and enjoyment were reported to be higher with IVR compared to the 2D screen. Additionally, interest and enjoyment with IVR were also higher than with AR, and AR exhibited a trend of higher interest and enjoyment compared to the 2D screen. | Evaluation:  The questionnaire is divided into six subjective subscales that target Mental and Physical.  Demand, Temporal Demand, Performance, Effort, and Frustration | 7/8 |
| Ventura, S. [4]  Department of Psychology, University of Bologna, Bologna, Italy  *Embodied the Healthy Arm: Virtual Reality Rehabilitation for Stroke Patients with Proprioceptive Upper-Limb Deficit*  Cyberpsychology, Behavior, and Social Networking | 2023 | The aim of the project is to investigate whether patients who embody a virtual arm with their injured one would induce the motor rehabilitation of the upper limb after a stroke. | 3 healthy subjects (2 male, one woman). | Upper limb, VR, FPP | HMD not specified, Khymeia SW. | The patients are invited to perform exergames (pinching, grasping, single fingers coordination) with the injured arm in two different sessions: IVR and non-VR. | After the rehabilitation, all patients improved their motor abilities, assessed by Fugl-Meyer, Motricity Index, Box, and Blocks.  In the VR sessions, patients perceive the virtual arm as their injured one. | Fugl Meyer, Motricity Index, Box, and Blocks assessment and evaluation. | 6/8 |
| Phelan, I. [5]  College of Social Sciences and Arts, Sheffield Hallam University  *Home‑based immersive virtual reality physical rehabilitation in pediatric patients for upper limb motor impairment: a feasibility study*  Virtual Reality | 2023 | This feasibility study aimed to explore the perceptions and impacts of an immersive and interactive VR scenario suitable for Upper limb motor impairment rehabilitation (ULMI) for children at home. | 8 subjects  (3 boys and 5 girls) | Upper limb, VR, FPP | Meta Quest. Unreal Engine 4.23, 3ds Max 2021, and Substance Designer 11.3. | The patients are invited to play a 3D exergames with two levels: Forest level acts as a tutorial for the archery mechanic, followed by the Tower to introduce climbing.  Children were then asked to use the IVR system for approximately 15 minutes twice a day at home for three weeks. | IVR for ULMI home rehabilitation could  be easy to learn and acceptable, improve motor function, reduce the difficulty in the reproduction of therapeutic movements. | Assessment and evaluation: - A goniometer (Standard BASELINE® 12-inch) was used by the physiotherapist to measure a Range of Movement (ROM) of the affected and unaffected upper limb joint (flexion, extension, abduction, and adduction) before and after the at-home trial. Paediatric Quality of Life Inventory (Version 4.0 – UK English) (PedsQL) is a standardized assessment for children aged 5–18 and was completed by patients before and after the IVR rehabilitation at-home trial.  System Usability Scale.  Semi structured interviews | 8/8 |
| Camardella, C. [6]  Institute of Mechanical Intelligene, Scuola Superiore Sant’Anna, Pisa  *Introducing wearable haptics for*  *rendering velocity feedback in VR serious games for neuro-rehabilitation of children*  Frontiers in Virtual Reality | 2023 | To show the design, implementation, and first evaluation of a gaming scenario for upper limb rehabilitation of children with cerebral palsy. | 8 healthy subjects. | Upper limb, VR, FPP | Oculus quest 2 | The VR environment depicts a magical training ground for wizards, surrounded by mountains and trees, with a large rune on the floor marking the spell-casting area. Players use their index finger as a wand to draw symbols in the air, casting spells at enemies. The accuracy of symbol drawing determines spell potency, with reference to a 2D sample provided. Two custom lightweight haptic thimbles provide tactile feedback. Conditions studied include speed reference presence and feedback type—3838haptic or visual—related to tracking velocity. | Preliminary examinations conducted on healthy participants revealed that the introduction of haptic feedback didn't notably change the perception of absolute speed or the capability to uphold a steady self-selected reference speed. Nevertheless, when participants were directed to adhere to a predetermined reference speed, the incorporation of haptic feedback improved performance by enhancing smoothness and diminishing speed-tracking errors. However, it's worth noting that only smoothness demonstrated statistically significant improvement. | Evaluation:  Analysis of the dataset built using the recorded hand speed as a feature. | 7/8 |
| Song, Z. [7]  School of Biological Science and Medical Engineering, Beihang University, Beijing  *The third-person perspective full-body illusion induced by*  *visual-tactile stimulation in virtual Reality for stroke patients*  Consciousness and Cognition | 2023 | To induce the third-person perspective full-body illusion (3PP-FBI) with virtual reality (VR) in stroke patients. | 19 post-stroke subjects (6 females, 13 males) | Full body, VR, TPP | Valve Index VR headset + controllers | Four experimental conditions were tested: synchronous visual-tactile stimulation on the back (Back-S), synchronous visual-tactile stimulation on the arm (Arm-S), non-synchronous visual-tactile stimulation on the back (Back-NS), and non-synchronous visual-tactile stimulation on the arm (Arm-NS). During the experiment, the experimenter randomly touched the participant’s back or arm with a physical bar. In synchronous conditions, virtual and physical bar stimulation matched temporally and spatially. | The findings demonstrated that virtual reality (VR) could trigger the sensation of ownership of a third-person perspective (3PP-FBI) in stroke patients, akin to how it does in healthy individuals, through synchronous visual-tactile stimulation of a specific body part (such as the back or upper limb).  Furthermore, it was observed that stimulating the back could evoke a more pronounced sense of 3PP-FBI compared to stimulating the affected upper limb. This suggests that for stroke patients experiencing limb dysfunction, stimulating the back may be more effective in inducing a robust sense of ownership from a third-person perspective. | Questionnaire scores reflect the subjective experience of the participants, and self-location drift values reflect the objective self-location perception. | 8/8 |
| Buetler K. [8]  Motor Learning and Neurorehabilitation Laboratory, ARTORG Center for Biomedical Engineering Research, University of Bern, Bern, Switzerland  *"Tricking the Brain" Using Immersive Virtual Reality: Modifying the Self-Perception Over Embodied Avatar Influences Motor Cortical Excitability and Action Initiation.*  Frontiers in Human Neuroscience | 2022 | Testing the adaptation of motor commands based on perceived modified reality, using a “Stone Arm Illusion.” | 10 healthy subjects | Upper arm, VR, FPP | HTC Vive with trackers. Unity 3D, The Black Box response buttons, Magstim 200 Mono Pulse stimulator. A TMS navigation system | The participants viewed in VR an avatar from a first-person perspective. The surface of the avatar was gradually transformed from human to stone. This visual change was reinforced by repeatedly touching the participant's real arm and the avatar's arm with a virtual hammer while progressively replacing the sound of the hammer hitting the skin with the sound of the hammer hitting a stone via a loudspeaker. TMS was used to evaluate changes in motor cortical excitability associated with the illusion. Additionally, to investigate if the “stone illusion” affected motor control, participants performed a reaching task with both the human and stone avatar. | Participants reported a complete immersion in the "stone arm illusion." Through immersive virtual reality (VR) and multisensory feedback, they perceived their arm as colder, heavier, stiffer, and less sensitive when they experienced illusory ownership over a stone arm compared to a human avatar. The extent of the stone illusion influenced participants' perception of their ability to control their arm, yet they consistently regarded the virtual stone arm as their own. | Two questionnaires to assess the subjectively reported embodiment and the perceptual correlates of the stone arm illusion.  Evaluation:  - Motor evoked potentials amplitudes  - kinematic variables data analysis. | 7/8 |
| Fregna, G. [9]  Univ Ferrara, Doctoral Program Translat Neurosci & Neurotechnol, Ferrara, Italy  *A novel immersive virtual reality environment for the motor rehabilitation of stroke patients: A feasibility study*  FRONTIERS IN ROBOTICS AND AI | 2022 | Testing the substantial subjective impressions of ownership of the virtual body. | 16 post stroke subjects | Upper arm, VR, FPP | Oculus Quest 2. Unity3D | The patients are immersed in a VR environment by means of a head-mounted display. In this environment, they can see different objects with which they can interact with a series of tasks. The program running on the HMD wirelessly communicates with a client app running on a remote PC. | Nearly all participants provided the highest rating for their experience, and in a standardized survey, they indicated a strong sense of possession of the virtual hands and control in the VR setting generated by those virtual hands. The findings demonstrate that using virtual hands resulted in significant subjective perceptions of owning the virtual body and having agency among the participants. | Assessment:  Fugl-Meyer Assessment for Upper Extremity  Evaluation:  - Subset of a standardized questionnaire proposed by Gonzalez-Franco and Peck (2018).  - A satisfaction questionnaire.  - Trajectories data analysis. | 8/8 |
| Pais-Vieira, C. [10]  Instituto de Ciências da Saúde (ICS), Universidade Católica Portuguesa, Porto, Portugal  *Embodiment Comfort Levels During Motor Imagery Training Combined with Immersive Virtual Reality in a Spinal Cord Injury Patient*  Frontiers in Human Neuroscience | 2022 | Testing level and comfort of embodiment experience. Reducing pain. | Single case study on a SCI (spinal cord injury) subject. | Lower limbs, VR, FPP | HTC Vive Pro Eye, custom-developed thermal-tactile sleeves with thermal stimulation, EEG Brain Products GmbH, Gilching, OpenVibe, Cycling 74 Max, | Each session consisted of three different phases: habituation, data acquisition, and real-time decoding. 1) During habituation,  the participant triggered each step of the avatar using the hand controller and received visual, auditory, and thermal-tactile feedback. 2) During the data acquisition phase, neural data was recorded while the subject performed the motor imagery task and received visual, auditory, and thermal-tactile feedback  3) The neural decoding phase was similar in all aspects to the data acquisition phase, with the exception that the classifier, trained with the data acquired in the second phase, would now decode in  real-time neural activity recorded from the participant. | Participants reported high levels of embodiment experiences and a reduction in pain, expressing comfort with the embodiment encounters. The EEG decoding performance averaged at 75 +/- 23%. | Comfort evaluation questionnaire  EEG Data analysis. | 8/8 |
| Wenk, N. [11]  Artorg Center for Biomedical Engineering Research, University of Bern  *Hiding Assistive Robots during Training in Immersive VR Does Not Affect Users' Motivation, Presence, Embodiment, Performance, Nor Visual Attention*  IEEE Signal Processing Letters | 2022 | This study explores how motivation, embodiment, and presence are impacted if neurorehabilitation robots are not displayed in VE during sessions. | 28 healthy subjects aged from 21 to 64. | Upper arm,  VR, FPP | BURT Upper-limb rehabilitation  end-effector robot.  HTC Vive  Pro Eye + trackers.  Unity 3D | Participants immersed in the virtual environment (VE) from a first-person perspective controlled an avatar's head and right arm. They were instructed to navigate paths quickly and accurately, collecting coins along the way using a sphere held by the avatar's hand, similar to the real sphere held by participants on the robot end-effector. Paths were defined by cubic Bezier curves scaled to the participant's workspace. Coins disappeared upon collection, accompanied by a sound effect. Participants completed two laps on each of the seven paths, aiming to improve their second lap time for applause. Path order was randomized, and a user interface provided lap time feedback. | Following each trial, participants were instructed to remove the head-mounted display (HMD) and complete a series of questionnaires. The authors documented the participants' gaze behaviour throughout the task. The authors did not observe a significant main effect of the robots' visibility in the virtual environment (VE). Additionally, no interaction effect was detected between visibility and the level of assistance provided by the robots. | Evaluation:  Questionnaires on presence, embodiment, and motivation.  Trajectories and gaze data analysis. | 8/8 |
| Sanford, S. [12]  Altorfer Complex Stevens Institute of Technology, Hoboken, NJ, United States  *Investigating features in augmented visual feedback for virtual reality rehabilitation of upper-extremity function through isometric muscle control*  Frontiers in Virtual Reality | 2022 | This study represents an initial exploration into the impact of different levels of complexity and intermittency in augmented visual guidance on the performance of an isometric muscle control task using a computerized platform. | 13 healthy participants (seven males, six females) | Upper arm,  VR, FPP | Position-adjustable brace that isometrically supports the upper arm. Trigno Wireless EMG System. g.USBamp EEG.  Shimmer3 GSR. Headset not specified. | This study utilized augmented training guidance through visual cues indicating deviations from the shortest path between starting positions and targets. A semi-transparent "ghost" robot avatar was displayed alongside the participant-controlled avatar during training, projecting the participant's movements onto the optimal path. Four modes of augmented visual feedback were created by varying complexity (amount of visual information) and intermittency (frequency of visual information), each tested at two levels. | When aiming to enhance upper-extremity function through training, emphasizing end-effector accuracy alone, without supplementary visual feedback for the forearm and upper arm, leads to improved motor performance and increased arousal. Training with simpler feedback led to shorter completion times and minimized path lengths, whereas intermittent feedback enhanced muscle-level control and decreased cognitive activity. These results imply that investing resources in physical engagement and performance yields favourable outcomes. | Evaluation:  EMG, EEG, EDA data analysis | 8/8 |
| Matsumiya, K. [13]  Tohoku Univ, Grad Sch Informat Sci  Japan  *Awareness of voluntary action,*  *rather than body ownership,*  *improves motor control*  SCIENTIFIC REPORTS | 2021 | To determine the functional roles of agency and body ownership in motor control. | 20 healthy subjects (7 women, 13 men; mean age 22.75 [range 19–28] years)  in experiment 1, 29 healthy subjects (13 women, 16 men; mean age 22.66 [range 19–28] years) in experiment 2. | Upper arm,  VR, FPP | HTC Vive, PHANToM force-feedback device, Eye tracker | A session consisted of a hand-movement task and a finger-tracking task. During the task, an eye tracker records eye movement.  In the passive condition, the participant's entire right arm, which was concealed from view, was moved by the arm of a force-feedback device attached to the participant's right index finger. In the synchronous condition, the CG hand moved in perfect sync with the participant's hand movements. In the asynchronous condition, whether the participant's hand was moved actively or passively, the CG hand movements were delayed by 0.5 seconds. Consequently, four conditions were defined: active-synchronous, active-asynchronous, passive-synchronous, and passive-asynchronous. | The research findings indicate that in the synchronous condition, both agency and motor commands were effective, whereas only agency appeared to be effective in the asynchronous condition. Specifically, participants reported a sense of agency over the CG hand in both the active-synchronous and active-asynchronous conditions. However, motor commands aligned with the visual feedback of the moving hand (i.e., the CG hand in motion) only in the active-synchronous condition, not in the active-asynchronous condition. | Evaluation:  Questionnaire  - Gaze analysis | 8/8 |
| Odermatt, I.A. [14]  Univ Bern, ARTORG Ctr Biomed Engn Res  *Congruency of Information Rather*  *Than Body Ownership Enhances*  *Motor Performance in Highly*  *Embodied Virtual Reality*  FRONTIERS IN NEUROSCIENCE | 2021 | To investigate the effect of body ownership and congruency of information on motor performance in immersive virtual reality, modulating body ownership by providing congruent vs. incongruent visuotactile stimulation. | 50 healthy subjects (35 females, 15 males) | Hand / Upper arm, VR, FPP | HTC Vive + controllers, 4-button response box. GSR g.Sensor, g.tec Medical Engineering  Unity 3D, MakeHuman | Participants received visuotactile stimulation during a simple task with the controllers and the buttons. Directly after the tasks, a threat, i.e., a virtual knife, fell from above the vision field and stabbed the virtual hand. GSR data were captured during the sessions. | The findings of the author indicate that virtual reality (VR) motor tasks, which offer consistent sensory feedback across multiple senses and promote a sense of body ownership and agency through visual-motor synchronizations, are most beneficial for motor training. Utilizing immersive VR from a first-person perspective could streamline the creation of effective training environments, particularly in the field of (robotic) neurorehabilitation. | Evaluation:  - Questionnaire  -GSR data analysis | 8/9 |
| Tambone, T. [15]  Department of Psychology, University of Turin  *Using Body Ownership to Modulate*  *the Motor System in Stroke Patients*  Psychological Science | 2021 | Testing body ownership illusion, confronting FP e TP perspective. | 12 subjects with left hemisphere damage and chronic motor deficits | Full body, VR, FPP and TPP | Oculus Rift CV1 (Oculus VR, Irvine, CA) equipped with two PenTile organic light-emitting diode displays.  Unity3D. | The experiment consisted of two scenes seen in VR in two different sessions, one in FPP (first-person perspective) and the other in TPP (third-person perspective). | According to a questionnaire, for the embodiment group (first-person perspective), the median score for the illusion question was significantly higher than the median score for the control question for ownership (third-person perspective) | Eight types of gait tests for assessment and post-trial evaluation.  Evaluation with questionnaire on the feelings of ownership and agency over the avatar on a Likert-type scale | 8/8 |
| Garcia-Hernandez N. [16]  Centre for Research and Advanced Studies of the National Polytechnic Institute (CINVESTAV-IPN), Saltillo, Mexico  *Virtual body representation for rehabilitation influences on motor*  *performance of cerebral palsy children*  Virtual Reality Volume 25 | 2021 | To examine how the subjective experience of seeing and controlling a half-body avatar, or an abstract hand representation in a moderate immersion virtual environment (VE), for training upper limb movements may affect CP children’s motor performance | 19 subjects with cerebral palsy (13 males, six females) | Upper limb, VR,TPP | KinectV2, 50’ flat screen (no headset), Unity3D | Children visualized a virtual scene from a third-person perspective, behind the avatar or abstract object. They have to reach three target balls located over their shoulder level and release them in a box below their shoulder level; four trials were performed with a TPP avatar and four with an abstract avatar (a sphere) | Humans improve their movement efficiency by reducing specific cost factors. Findings indicate that the virtual representation of the body notably impacts task performance indicators and cost factors associated with upper limb movements, especially in reaching tasks as opposed to releasing tasks. When children with cerebral palsy (CP) visualize hand movements using an abstract object, they complete tasks 22% faster, with 28% less overall movement, and exhibit 62% fewer jerky movements compared to visualizing the entire arm through a realistic avatar. | Evaluation:  Movements data analysis | 8/8 |
| Matamala-Gomez, M. [17]  University of Milano-Bicocca  *Changing Body Representation Through Full Body Ownership Illusions Might Foster Motor Rehabilitation Outcome in Patients with Stroke*  FRONTIERS IN PSYCHOLOGY | 2020 | Assessment and modulation of the internal representation of the affected upper limb in stroke patients | Not specified | Upper limb, VR, FPP | Not specified | Patients will observe the virtual body (360 VIDEO) that will be collocated with their real body and will represent the patient’s described distorted representation of the upper limb from a first-person perspective. Then, they observe the progressive transformation of the affected upper limb from a distorted representation to a normal one. | Not specified | Not specified | 7/8 |
| Heinrich, C. [18]  Department of Information Science, University of Otago, Dunedin, New Zealand  *My hands? Importance of personalized virtual hands*  *in a neurorehabilitation scenario*  Virtual Reality | 2020 | To demonstrate that the higher realism of virtual hands achieved (AV, Augmented virtuality) by a novel texturing approach alters perceived embodiment. | 48 healthy subjects | Hands, VR, FPP | Oculus Rift CV1.  Leap Motion | In the first experiment, to test the AV hands, participants interacted with two randomly assigned hand visualizations and experienced both mirrored and non-mirrored conditions for each. In a second experiment, the authors wanted to investigate how participants perceived their own hand size in a virtual environment by allowing them to resize the virtual hand model to what they perceived to be their real hand size. | The findings from the questionnaires indicate that healthy users can experience a strong sense of embodiment with virtual hands in augmented reality (AV). Additionally, the second study illustrates that the size of the virtual hands in AV environments has minimal to no impact on this sense of embodiment. | Evaluation:  - Hand Visualisation Realism Questionnaire  - Embodiment Virtual Reality Questionnaire (Lin and Jörg adaptation, 2016) | 8/8 |
| Lim, H. [19]  Keimyung University, Daegu, Republic of Korea  *Transcranial Direct Current Stimulation Effect on Virtual Hand Illusion*  Cyberpsychology, behavior and social networking | 2020 | To investigate whether a mirror virtual hand illusion could be modulated by tDCS | 14 healthy subjects (7 male, 7 female) | Hand, VR,FPP | tCDS stimulator, Wearable Sensing DSI-24 EEG. Headset not specified. | Virtual mirror tasks are conducted with and without tCDS on two different days. In the task, participants flex their right arm while seeing the corresponding movement in the left hand. | A more pronounced proprioceptive change occurred during tasks involving tCDS, indicating support for employing the integrated system in the rehabilitation of post-stroke patients. | Evaluation:  - EEG data analysis.  - Questionnaire about ownership and control of the mirrored virtual hand. | 7/8 |
| Kaneko F. [20]  Keio Univ, Sch Med, Dept Rehabil Med, Tokyo, Japan  *A Case Series Clinical Trial of a Novel Approach Using Augmented Reality That Inspires Self-Body Cognition in Patients with Stroke: Effects on Motor Function and Resting-State Brain Functional Connectivity.*  Frontiers in Systems Neuroscience | 2019 | This study aimed to clarify the effect of the kinesthetic perception illusion induced by visual stimulation (KINVIS) on upper limb motor function and the relationship between motor function and resting-state brain networks. | 11 subjects with severe paralysis in the upper limbs (4 women). | Upper limb, AR, FPP | Augmented reality system using monitors. fMRI is used to evaluate motor functions and resting-state brain functional connectivity. | The cognitive phenomenon of KINVIS can be described as the feeling of one’s body moving during sensory input, even though the body is actually in a resting state.  The subjects were applied visual stimulation for 20 min together with neuromuscular electrical stimulation. The patients were seated at a chair with their forearms on the table. The hand movement of the unaffected side was recorded before the intervention. The movement task involved hand opening and closing. This task was executed using the unaffected side and was flipped to mirror the movement of the affected side. | The results of motor function (Fugle-Meyer Assessment, FMA) and spasticity (Modified Ashworth Scale, MAS) showed significant improvement following the intervention. | Assessment and evaluation:  - Fugl-Meyer assessment,  - Modified Ashworth Scale,  - Action Research Arm test,  - Box and Block Test,  - Motor Activity log,  - MRI data analysis. | 8/8 |
| Borrego A. [21]  Instituto de Investigación e Innovación en Bioingeniería, Universitat Politècnica de València  *Embodiment and Presence in Virtual Reality After Stroke. A Comparative Study with Healthy Subjects*  Frontiers in Neurology | 2019 | To determine and compare the sense of embodiment and presence elicited by a virtual environment under different perspectives and levels of immersion in healthy subjects and individuals with stroke | 46 healthy subjects (25 male + 21 female) and 32 subjects with Stroke (18 male + 14 female) | Full body, VR, FPP and TPP | Oculus Rift CV1, Microsoft Kinect | The VE consisted of an infinite checkered floor, with a central grey circle and a gender-matched mesomorph avatar, which synchronously mimicked the participants’ movements. Playdough-coloured items (cubes, spheres, and cones) appeared on the floor in front of the central circle.  The objective of the task was to step on the items before they disappeared with the closest avatar foot while keeping the other foot inside the central circle. In between stepping on the items, the foot used had to be moved back into the circle.  The task has to be performed in two modes: FPP and TPP (screen) | Consistently higher levels of embodiment and presence were observed in healthy subjects when using the first-person perspective. These findings highlight that utilizing a first-person perspective through a head-mounted display (HMD) induced a stronger feeling of body ownership and self-location compared to embodying a virtual avatar, leading to an enhanced sense of presence across both populations. However, the sense of agency remained relatively consistent across different conditions. Notably, participants with stroke consistently reported less immersive experiences compared to their healthy counterparts. | Evaluation:  - Adapted version of the Embodiment of Rubber Hand Questionnaire.  Slater-  - Usoh Steed Questionnaire. | 8/9 |
| Caola, B. [22]  University of East London  Italy  *The bodily illusion in adverse conditions: virtual arm ownership during visuomotor mismatch*  Perception | 2018 | Exploring whether it is possible to induce a sense of body ownership over a virtual body part during visuomotor inconsistencies, with or without the aid of concomitant visuotactile stimulations. | 45 healthy subjects (22 female) | Upper limb, VR, FPP | Oculus Rift DK2.  Unity 3D A small vibrator was placed in the middle of the right participant’s hand dorsum and controlled via an Arduino board. | From a first-person perspective, the participant watched a virtual tube moving or an avatar’s arm moving, with or without concomitant synchronous visuotactile stimulations on their hand. Three different virtual arm/tube speeds were also investigated, while all participants kept their real arms still. | The primary discovery from this research is that it's feasible to generate a feeling of ownership over a virtual body part, even in the presence of a significant discrepancy between the real and virtual limb's visual and motor aspects. These findings were derived from a questionnaire-based approach. | Evaluation:  Questionnaire  (Slater, Perez-  Marcos, Ehrsson, & Sanchez-Vives, 2008). | 7/8 |
| Inamura, T. [23]  Natl Inst Informat, Principles Informat Res Div, Tokyo, Japan  *Development of VR platform for cloud-based*  *neurorehabilitation and its application to research*  *on sense of agency and ownership*  ADVANCED ROBOTICS | 2017 | To determine the feasibility of the VR system – specifically, whether it has enough effect on sense of agency (SoA) and sense of ownership (SoO) for healthy subjects – before conducting experiments for actual phantom limb patients | 12 subjects (8 males, 2 Females) | (Phantom) Limb, VR, FPP | SIGVerse system.  Oculus Rift DK2  Kinect V2 | VR system in which a virtual avatar performs a motion identical to that of the subject by means of a motion-capturing device. The subject wears a 3D head-mounted display to experience seeing through the eyes of the avatar. Six conditions of avatar representation were used: two appearances of a normal human arm and a robot arm and three lengths of the arm (short, medium, and long). The subject executes elbow flexion-extension movement of the right arm, which causes the same movement in the VR avatar’s arm. | The findings indicated that the perceived length of the arm was altered based on the displayed arm's length within the virtual reality (VR) setting. Through analysis of questionnaire responses, it was determined that there was no adverse impact on the Sense of Agency (SoA). Additionally, it was observed that the Sense of Ownership (SoO) was stronger when participants viewed a natural human avatar compared to when they observed a robot arm. | Evaluation:  - Questionnaire.  - Gesture data analysis (after the induction movement, the subjective sense of the length of the right arm is measured by  a pointing gesture of the left hand.) | 7/8 |
| Pozeg, P. [24]  École Polytechnique Fédérale de Lausanne, Campus Biotech, Geneva, Switzerland  *Virtual reality improves embodiment and*  *neuropathic pain caused by spinal cord*  *injury*  Neurology | 2017 | To investigate changes in body ownership and chronic neuropathic pain in patients with spinal cord injury (SCI) using multisensory own body illusions and virtual reality (VR). | 20 subjects with SCI with paraplegia and 20 healthy subjects | Legs, VR, FPP and TPP | Not specified | Virtual leg illusion + full-body illusion, using asynchronous or synchronous visuotactile stimulation.  The HMD shows the real-time (or delayed) video of dummy legs from the distance and angle that corresponds to the participant’s first-person viewpoint. | The findings reveal that individuals with Spinal Cord Injury (SCI) exhibit reduced sensitivity to multisensory stimuli that generate the illusion of leg ownership in comparison to those without such injuries (HC). Furthermore, the sense of leg ownership diminished over time following SCI. Interestingly, there were no discernible distinctions between the groups in terms of overall body ownership, as assessed by the FBI. | Assessment and evaluation:  - VLI was assessed with a 9-item questionnaire  adapted from body illusions studies.  - The FBI was assessed with a 7-item questionnaire.  actual neuropathic pain has been assessed  with a visual analog scale | 9/9 |
| Shokur, S. [25]  AASDAP, Neurorehabil Lab, Sao Paulo, Brazil  *Assimilation of virtual legs and*  *perception of floor texture by*  *complete paraplegic patients*  *receiving artificial tactile feedback*  Scientific Reports | 2016 | Reproducing lower limb somatosensory feedback in paraplegics by remapping missing leg/foot tactile sensations onto the skin of the patient’s forearms. | 7 subjects with SCI | Legs, VR, FPP | HMD not specified. “Tactile shirt” with eccentric mass (ERM) vibrators to deliver somatosensory feedback | Immersive virtual reality system where the subjects’ lower limbs were simulated by a human-like 3D avatar seen in FPP with a HMD. Tactile and proprioceptive sensations generated by the avatar’s virtual legs were mapped on the patients’ forearms by means of arrays of vibrators that defined a haptic display.  Two months later, a second session without feedback was administered. | After the experiment, six out of eight patients reported in a questionnaire that they experienced again the vivid sensation of walking on the three chosen ground surfaces. | Evaluation:  - Questionnaire. - Analyse the experiment data (keypress timings, performance score, etc.) | 8/8 |
| Llobera, J. [26]  EVENT Lab, University of Barcelona, Barcelona  *Virtual reality for assessment of patients suffering chronic pain:*  *a case studies*  Experimental Brain Research | 2013 | The case study wants to show that the induction of virtual body ownership combined with simple electrophysiological measures could be useful for the diagnosis of patients with neurological conditions. | A case study on a subject with fixed dystonia, + 5 controls | Upper and lower limbs, VR, FPP+TPP | NVIS SX111 HMD. InterSense IS900 head tracker.  EMG+EEG gUSBAmp. 6-degrees-of-freedom Wand device for visuotactile experience. | Authors propose a method that exploits ownership of a virtual body in combination with a simple brain-computer interface (BCI) and basic physiological measures to complement neurological assessment.  Steps of the trial:  (a) Induction of body ownership.  (b) Opening of the virtual hand using BCI  (c) Reducing the size of the ball using BCI  (d) Repeat (b).  (e) Repeat (c).  The EMG was measured throughout to assess the impact of the visual feedback on the muscular activity of the real hand. | Despite the small scope of this particular case study, the findings indicate that the sense of owning a virtual body, combined with basic electrophysiological equipment such as a single-surface EMG electrode and a single electrode BCI, can be utilized to evaluate movement-related disorders in neurological examinations. | Assessment and evaluation:  - EMG and EEG data analysis.  - Questionnaire on body ownership. | 8/9 |

## Reference List for Multimedia Appendix 1

1. Dong Y, et al. A haptic-feedback virtual reality system to improve the Box and Block Test (BBT) for upper extremity motor function assessment. Virtual Reality. 2023.
2. Batista D, et al. Effect of head-mounted virtual reality and vibrotactile feedback in ERD during motor imagery brain–computer interface training. Brain–Computer Interfaces. 2023.
3. Wenk N, et al. Effect of immersive visualization technologies on cognitive load, motivation, usability, and embodiment. Virtual Reality. 2023.
4. Ventura S, et al. Embodied the healthy arm: Virtual reality rehabilitation for stroke patients with proprioceptive upper-limb deficit. Cyberpsychology, Behavior, and Social Networking. 2023.
5. Phelan I, et al. Home-based immersive virtual reality physical rehabilitation in pediatric patients for upper limb motor impairment: A feasibility study. Virtual Reality. 2023.
6. Camardella C, et al. Introducing wearable haptics for rendering velocity feedback in VR serious games for neuro-rehabilitation of children. Frontiers in Virtual Reality. 2023.
7. Song Z, et al. The third-person perspective full-body illusion induced by visual–tactile stimulation in virtual reality for stroke patients. Consciousness and Cognition. 2023.
8. Buetler K, et al. "Tricking the brain" using immersive virtual reality: Modifying the self-perception over embodied avatar influences motor cortical excitability and action initiation. Frontiers in Human Neuroscience. 2022.
9. Fregna G, et al. A novel immersive virtual reality environment for the motor rehabilitation of stroke patients: A feasibility study. Frontiers in Robotics and AI. 2022.
10. Pais-Vieira C, et al. Embodiment comfort levels during motor imagery training combined with immersive virtual reality in a spinal cord injury patient. Frontiers in Human Neuroscience. 2022.
11. Wenk N, et al. Hiding assistive robots during training in immersive VR does not affect users' motivation, presence, embodiment, performance, nor visual attention. IEEE Signal Processing Letters. 2022.
12. Sanford S, et al. Investigating features in augmented visual feedback for virtual reality rehabilitation of upper-extremity function through isometric muscle control. Frontiers in Virtual Reality. 2022.
13. Matsumiya K, et al. Awareness of voluntary action, rather than body ownership, improves motor control. Scientific Reports. 2021.
14. Odermatt IA, et al. Congruency of information rather than body ownership enhances motor performance in highly embodied virtual reality. Frontiers in Neuroscience. 2021.
15. Tambone T, et al. Using body ownership to modulate the motor system in stroke patients. Psychological Science. 2021.
16. Garcia-Hernandez N, et al. Virtual body representation for rehabilitation influences on motor performance of cerebral palsy children. Virtual Reality. 2021.
17. Matamala-Gomez M, et al. Changing body representation through full body ownership illusions might foster motor rehabilitation outcome in patients with stroke. Frontiers in Psychology. 2020.
18. Heinrich C, et al. My hands? Importance of personalized virtual hands in a neurorehabilitation scenario. Virtual Reality. 2020.
19. Lim H, et al. Transcranial direct current stimulation effect on virtual hand illusion. Cyberpsychology, Behavior, and Social Networking. 2020.
20. Kaneko F, et al. A case series clinical trial of a novel approach using augmented reality that inspires self-body cognition in patients with stroke: Effects on motor function and resting-state brain functional connectivity. Frontiers in Systems Neuroscience. 2019.
21. Borrego A, et al. Embodiment and presence in virtual reality after stroke: A comparative study with healthy subjects. Frontiers in Neurology. 2019.
22. Caola B, et al. The bodily illusion in adverse conditions: Virtual arm ownership during visuomotor mismatch. Perception. 2018.
23. Inamura T, et al. Development of VR platform for cloud-based neurorehabilitation and its application to research on sense of agency and ownership. Advanced Robotics. 2017.
24. Pozeg P, et al. Virtual reality improves embodiment and neuropathic pain caused by spinal cord injury. Neurology. 2017.
25. Shokur S, et al. Assimilation of virtual legs and perception of floor texture by complete paraplegic patients receiving artificial tactile feedback. Scientific Reports. 2016.
26. Llobera J, et al. Virtual reality for assessment of patients suffering chronic pain: A case study. Experimental Brain Research. 2013.
